# Editorial: Immunometabolism at the intersection of signaling networks and therapeutic strategies

**DOI:** 10.3389/fimmu.2026.2010979

**Published:** 2026-10-06

**Authors:** Luis Eduardo Alves Damasceno, Juliana Escher Toller-Kawahisa

**Affiliations:** 1 Department of Pharmacology, Ribeirao Preto Medical School, University of Sao Paulo, Ribeirao Preto, Brazil; 2 Center for Research in Inflammatory Diseases, Ribeirao Preto Medical School, University of Sao Paulo, Ribeirao Preto, Brazil; 3 School of Biochemistry and Immunology, Trinity Biomedical Science Institute, Trinity College Dublin, Dublin, Ireland

**Keywords:** autoimmunity, cancer, immune homeostasis, infection, inflammation, metabolic reprogramming, signaling pathways

This Research Topic highlights recent advances in the molecular and cellular mechanisms underlying metabolic-immune crosstalk across aging, inflammation, obesity, cancer, and neurological disease. Collectively, the studies demonstrate how metabolic pathways and their regulatory metabolites shape innate and adaptive immune function by integrating cellular bioenergetics with signaling, stress responses, and tissue-specific cues. In this context, the identification of key metabolic regulators and metabolites provides important insights into the mechanisms governing immune-cell adaptation and inflammatory responses. Furthermore, the papers published under this Research Topic emphasize metabolic remodeling as a potential therapeutic target, supporting the development of strategies to selectively modulate immune function across diverse pathological settings.

Recent studies have expanded our understanding of the interplay between mitochondrial homeostasis, immune function, and aging, highlighting complementary mechanisms through which mitochondrial quality control and metabolic adaptation shape immune responses. Ferreira et al. reviewed key mitochondrial proteases, including CLPXP, LONP1, m-AAA, i-AAA/YME1L, and OMA1, emphasizing that their functions extend beyond the removal of damaged proteins to include the regulation of mitochondrial dynamics, ROS production, cellular stress signaling, and mitochondria-derived danger signals, thereby modulating immune responses in a context-dependent manner. Similarly, Lewis et al. proposed that effective adaptation to mitochondrial stress through the mitochondrial unfolded protein response (mtUPR) is important for maintaining T_reg_ metabolic fitness and suppressive function during aging. Consistent with this framework, Queiroz et al. demonstrated in senescence-accelerated SAMP8 and senescence-resistant SAMR1 mice that metabolic and mitochondrial pathways can be pharmacologically modulated during aging. Six months of intermittent low-dose rapamycin increased hepatic mitochondrial respiratory capacity and ATP production while inducing strain- and tissue-specific changes in immune parameters, underscoring how modulation of nutrient-sensing pathways can alter mitochondrial function and immune phenotypes in the context of aging. Collectively, these findings support an interconnected network of mitochondrial proteostasis, stress responses, and nutrient-sensing pathways that regulates immune function during aging. Importantly, enhanced mitochondrial function does not necessarily translate into uniformly beneficial immune outcomes, highlighting the context dependence that should be considered when therapeutically targeting mitochondrial pathways.

Emerging evidence further highlights the importance of immunometabolic regulation in shaping immune function and inflammation during sepsis, a life-threatening condition characterized by a dysregulated response to infection that can lead to organ dysfunction and death. In this scenario, Xian et al. provide a focused overview of how systemic metabolic disturbances can translate into T-cell dysfunction, particularly through mitochondrial impairment and dysregulation of nutrient-sensing pathways. Their review highlights the roles of HIF-1α, mTOR, and AMPK in regulating T-cell exhaustion and the persistent immunosuppressive state associated with sepsis. This framework is extended to the tissue and organismal levels by Sui et al., who highlight that mitochondrial dysfunction is a central node linking immune dysregulation to oxidative stress, ferroptosis, and organ injury, with involvement of the gut-lung axis. Together, these studies support the concept that metabolic and mitochondrial alterations are not merely secondary consequences of sepsis but active determinants of immune dysfunction and tissue damage.

The metabolic plasticity of myeloid cells is also increasingly recognized as an important determinant of immune function across physiological and pathological settings. Integrating bulk and single-cell transcriptomic analyses with an experimental model of nephritis, Dai et al. showed that renal monocytes undergo substantial metabolic remodeling, characterized by enhanced steroid biosynthesis and a shift toward an M1-like phenotype mediated by the PI3K/Akt–SHIP1 axis. In a contrasting physiological context, Merech et al. demonstrated that pregnancy can induce metabolic adaptations that enhance macrophage-mediated clearance. Rather than simply reflecting a pro-inflammatory state, these metabolic changes appear to support specialized immune functions required during pregnancy. These findings emphasize that metabolic remodeling should not be interpreted exclusively as a signature of inflammation, but rather as a flexible process that integrates environmental cues with the functional state of immune cells.

The crosstalk between systemic and cellular metabolic cues and immune responses is further illustrated by studies spanning multiple levels of immunometabolic regulation, from endocrine and systemic factors to tissue-specific metabolic remodeling and discrete metabolic pathways that directly influence inflammatory signaling. Smith et al. discuss how glucocorticoids, estrogens, androgens, and progestogens regulate glycolysis, the tricarboxylic acid (TCA) cycle, and oxidative phosphorylation, thereby shaping immune-cell metabolism and inflammatory responses in a context-dependent manner. In obesity, these systemic metabolic influences are accompanied by profound remodeling of adipose tissue macrophage metabolism, particularly mitochondrial metabolism, which contributes to chronic inflammation and metabolic dysfunction, as reviewed by Jia et al. At the level of specific metabolic pathways, Qian et al. discuss the role of nucleotide metabolism in obesity-associated cardiovascular inflammation, illustrating how alterations in cellular metabolism can intersect with mitochondrial stress and innate inflammatory signaling. Collectively, these studies reinforce the concept that immunometabolism operates across multiple interconnected scales, in which systemic metabolic signals, tissue microenvironments, mitochondrial function, and specific metabolic pathways converge to shape immune-cell states and inflammatory responses.

A similar principle becomes particularly evident in cancer, as cancer metabolism functions as a dynamic signaling network through which immune cells adapt to and shape the tumor microenvironment. In fact, Frossi et al. position mast cells within this metabolic landscape, highlighting the bidirectional influence between the tumor microenvironment and mast-cell activity, including the potential effects of mast-cell-derived metabolites on tumor and stromal cells. Ye et al. further extend this concept to epigenetic regulation by proposing a “lactylation-immunosuppression network”, in which tumor-derived lactate functions not simply as a metabolic by-product but as a signaling metabolite capable of inducing both persistent histone-dependent transcriptional programs and rapid non-histone modifications that reinforce immunosuppressive states. Complementing these findings, Zotta further discusses the role of the mitochondrial electron transport chain in tumor-associated macrophages, demonstrating that individual respiratory complexes can differentially regulate cytokine production, macrophage function, and antitumor immunity, with preclinical evidence indicating that modulation of mitochondrial pathways may influence tumor control and response to immune checkpoint blockade. Together, these studies demonstrate that tumor metabolic organization is intrinsically linked to immune function and that this relationship is context-dependent. The emerging challenge is not only to target cancer metabolism, but also to elucidate and selectively modulate the metabolic interactions that connect malignant cells with the surrounding immune microenvironment.

The same immunometabolic principles observed in cancer are also evident in chronic inflammatory diseases, which can be conceptualized as prolonged inflammatory states with pathological consequences. Hu et al. apply this framework to inflammation-associated fatigue, proposing that persistent immune activation, mitochondrial dysfunction, and epigenetic acylation converge on metabolic and neuroimmune dysregulation that may contribute to fatigue across ME/CFS, long COVID, autoimmune disease, and cancer. In rheumatoid arthritis, Xu et al. similarly describe a self-reinforcing network linking inflammatory signaling with metabolic reprogramming, microbiota-derived metabolites, and extracellular-matrix remodeling. Together, these studies support a model in which reciprocal interactions among metabolic, signaling, epigenetic, and biomechanical pathways contribute to the persistence and propagation of chronic inflammation, shifting the focus from individual mediators toward the regulatory networks that maintain inflammatory states.

A similar interaction between metabolism and inflammation is also evident in the nervous system, where metabolic adaptation can critically influence responses to injury and infection. Rodrigues et al. describe how neurotropic viruses – including SARS-CoV-2, ZIKV, and HIV-1 – can reprogram microglial and astrocytic metabolism, often shifting cellular bioenergetics toward glycolysis, thereby linking mitochondrial dysfunction, altered metabolite availability, inflammasome activation, and sustained production of inflammatory mediators. Importantly, this review emphasizes the bidirectional relationship between metabolism and inflammation, in which components such as the NLRP3 inflammasome can both respond to metabolic stress and further reinforce a pro-inflammatory metabolic state. In a different but conceptually complementary setting, Wang et al. provide experimental evidence that pretreatment with Buyang Huanwu Decoction attenuates hypobaric hypoxia-induced brain injury in mice, improving behavioral and hippocampal outcomes while modulating HIF-1α, IL-6, VEGFA, NFκB1, and STX1A. Read together, these studies suggest that diverse neurological insults – whether initiated by viral infection or oxygen deprivation – may converge on a common immunometabolic vulnerability in which disturbances in cellular energy metabolism, hypoxic signaling, mitochondrial function, and innate immune activation become mutually reinforcing.

In summary, this Research Topic highlights immunometabolism as an interconnected and context-dependent regulatory network in which metabolic and signaling pathways converge to shape immune function across diverse physiological and pathological conditions (**Figure 1**). This complexity emphasizes the limitations of therapeutically targeting individual metabolic pathways in isolation. Future research should elucidate how these pathways regulate immune function and identify strategies to selectively modulate them while preserving physiological immunity.

**Figure 1:**
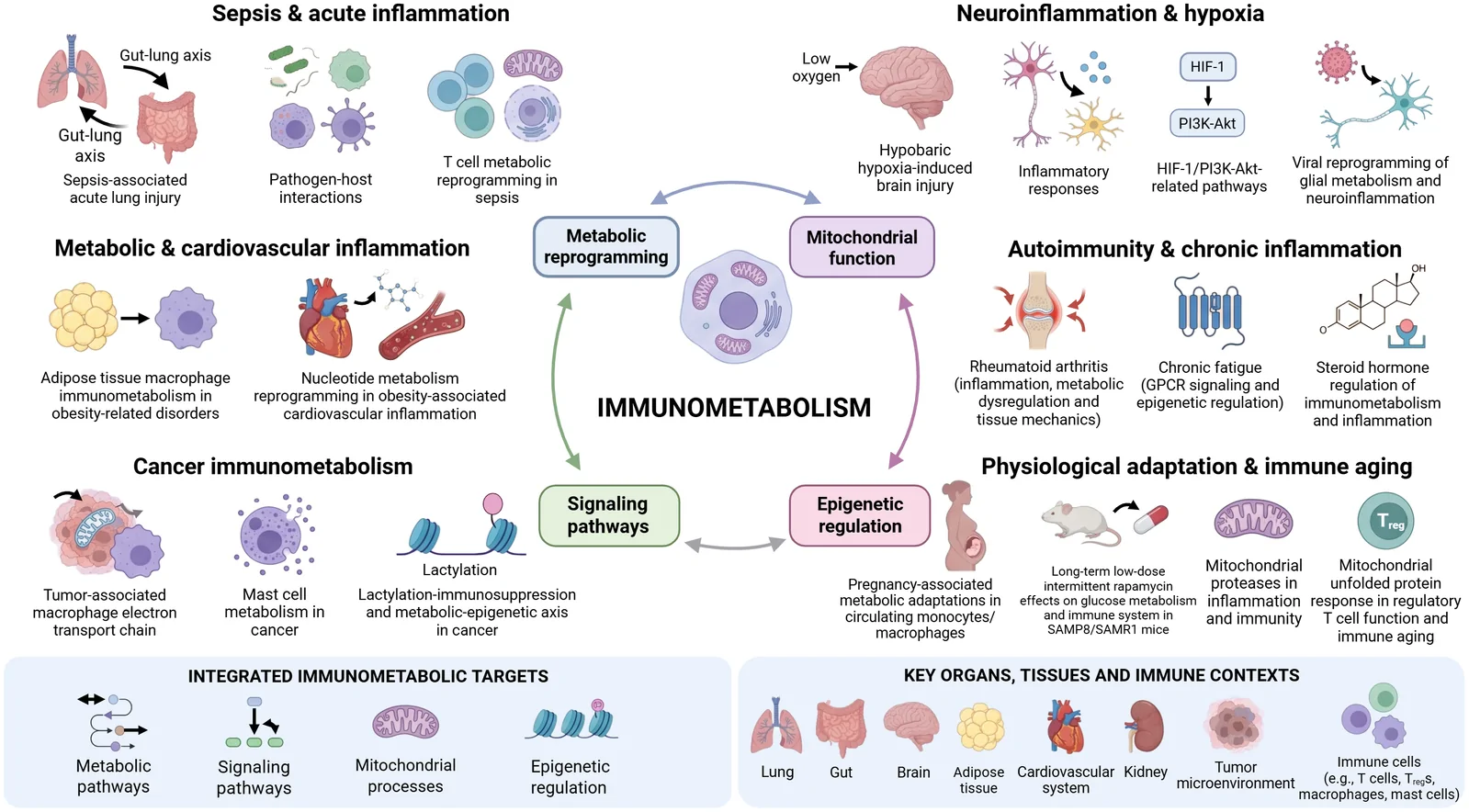
Overview of the interplay between metabolic and signaling pathways across the diverse physiological and pathological contexts explored in this Research Topic.

